# Variation in structure of proteins by adjusting reactive oxygen and nitrogen species generated from dielectric barrier discharge jet

**DOI:** 10.1038/srep35883

**Published:** 2016-10-25

**Authors:** Ji Hoon Park, Minsup Kim, Masaharu Shiratani, Art. E. Cho, Eun Ha Choi, Pankaj Attri

**Affiliations:** 1 Plasma Bioscience Research Center/Department of Electrical and Biological Physics, Kwangwoon University, Seoul 01897, Korea; 2 Department of Bioinformatics, Korea University, Sejong 02841, Korea; 3 Graduate School of Information Science and Electrical Engineering, Kyushu University, Fukuoka, Japan

## Abstract

Over the last few years, the variation in liquid chemistry due to the development of radicals generated by cold atmospheric plasma (CAP) has played an important role in plasma medicine. CAP direct treatment or CAP activated media treatment in cancer cells shows promising anticancer activity for both *in vivo* and *in vitro* studies. However, the anticancer activity or antimicrobial activity varies between plasma devices due to the different abilities among plasma devices to generate the reactive oxygen and nitrogen species (RONS) at different ratios and in different concentrations. While the generation of RONS depends on many factors, the feeding gas plays the most important role among the factors. Hence, in this study we used different compositions of feeding gas while fixing all other plasma characteristics. We used Ar, Ar-O_2_ (at different ratios), and Ar-N_2_ (at different ratios) as the working gases for CAP and investigated the structural changes in proteins (Hemoglobin (Hb) and Myoglobin (Mb)). We then analyzed the influence of RONS generated in liquid on the conformations of proteins. Additionally, to determine the influence of H_2_O_2_ on the Hb and Mb structures, we used molecular dynamic simulation.

The number of publications related to the biomedical application of cold atmospheric plasma (CAP) is rapidly increasing, demonstrating the vast potential and rapid evolution of the interdisciplinary field of biomedicine. It has been demonstrated that CAP can efficiently inactivate multidrug resistant microorganisms^1^,^2^,^3^,^4^. CAP is therefore extremely appropriate for the sterilization of immobilized microorganisms on a material surface under dry and wet conditions, as well as for a wide variety of other practical applications^5^,^6^,^7^,^8^. Additionally, CAP shows promising anticancer activities through apoptosis and cell cycle arrest, which have been shown to lead to the destruction of tumor cells in both *in vitro* and *in vivo* experiments^9^,^10^,^11^,^12^,^13^. Both anticancer activity and the inactivation of microbes by CAP are due to the generation of plasma agents such as photons, electric field, charged particles, and radicals, all of which have different effects on all biomolecular systems. In order to induce a specific biological effect using single or repeated plasma exposure, all reaction channels that can be triggered by the plasma action need to be explored, including not only simple physical or chemical damage to biomolecules but also soft structural modifications and changes in the biological environment, e.g. acidification or local reactive oxygen and nitrogen species (RONS) balance shift. However, the CAP efficiency needs to be improved for sterilization and the selectivity of CAP for cancer cells. This study provides an opportunity to modulate the RONS in liquid that can provide alternative possibilities to increase the efficiency of the CAP and improve its selectivity.

Cheng *et al*.^13^ treated normal human astrocytes (E6/E7) and glioblastoma cells (U87) to determine the threshold of plasma treatment while varying the gas composition of the plasma and varying the physical characteristics of plasma such as treatment time, flow-rate, and voltage. They observed that the He-O_2_ mixture weakened the therapeutic effect of cold plasma on cancer cells. Additionally, the cell viability increased by over 60% after 72 h, whereas the treatment of cells with He gas plasma jet showed only 30% increase in cell viability. In another study, oxygen addition to Ar plasmas has been shown to have an important effect on plasma sterilization efficiencies, which contradicts the above study, in which the addition of oxygen decreases the efficiency^14^. They found that the O_2_ addition to Ar plasma greatly improved the sterilization potential with the use of filter paper as the supporting medium, while the sterilization efficiency decreased in the presence of nutrient broth as the supporting medium. Hence, the effect of oxygen addition on the main working gas plasma depends on the physical characterization of plasma as well as the treatment conditions. Therefore, to understand the chemistry of mixed gases and the mechanism of action, we used Ar, Ar-O_2_ [0.2%, 0.3%, and 0.4% of O_2_ addition to Ar gas], and Ar-N_2_ [0.2%, 0.3%, and 0.4% of N_2_ addition to Ar gas] as feeding gases for the dielectric barrier discharge (DBD) plasma jet to treat the hemoglobin (Hb) and myoglobin (Mb) proteins at different time intervals. The generations of RONS from the mixed gases in the gas phase were studied using optical emission spectroscopy (OES), while the changes in RONS (OH radicals, NO radicals, H_2_O_2_, NO_2_^−^, and NO_3_^−^) in deionized (DI) water were studied using chemical and electrochemical analysis. Additionally, we checked the change in pH and temperature of the composition of mixed gases. Later, we studied the structural changes of Hb and Mb using circular dichroism, fluorescence spectroscopy, and Fourier transform infrared (FTIR) spectroscopy. We also performed the molecular dynamic simulation of Hb and Mb in the presence of H_2_O_2_ in order to understand the thermodynamics of both proteins in the presence of H_2_O_2_.

## Results

In our previous work, we reported the action of soft plasma and nanosecond pulsed plasma on the structural changes of Hemoglobin (Hb) and Myoglobin (Mb)^15^,^16^. However, until now, no report has been presented where the mixed gases plasma in different ratios is used to study the structural changes of proteins. Therefore, in this study, we used the dielectric barrier discharge (DBD) plasma jet with Ar, Ar-O_2_ (addition of O_2_ at different ratios to Ar gas), and Ar-N_2_ (addition of N_2_ at different ratios to Ar gas) as the working gas and treated model proteins (Hb and Mb) at different time intervals (1, 2, and 4 min).

### Reactive oxygen and nitrogen (RONS) species produced by mixed gas plasmas in gas phase

**Figure 1:**
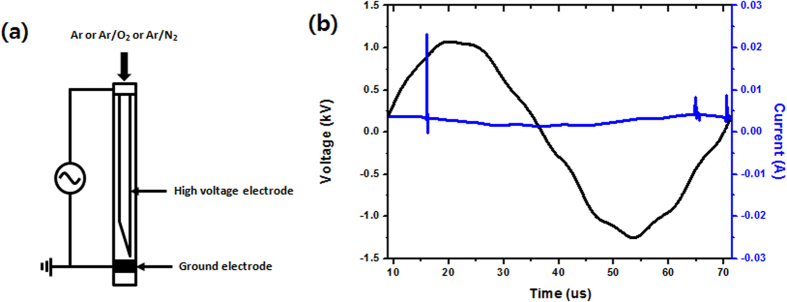
(**a**) Schematic diagram of the dielectric barrier discharge plasma (DBD) jet and (**b**) current-voltage curve of the discharge of Ar plasma.

**Figure 2:**
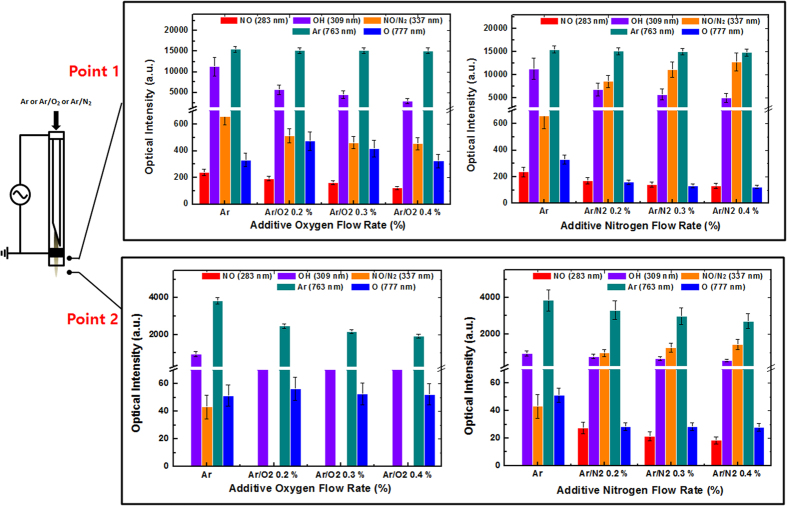
Radicals generated in gas phase using Ar, Ar-O_2_ mixtures, and Ar-N_2_ mixtures.

To understand the RONS produced by Ar, Ar-O_2_ [addition at 0.2%, 0.3%, and 0.4% O_2_ in Ar gas], and Ar-N_2_ [addition at 0.2%, 0.3%. and 0.4% N_2_ in Ar] as feeding gas plasmas, we used optical emission spectroscopy (OES). Figure 1a,b show a schematic representation of the plasma jet and the current voltage graph, respectively. The OES spectrum for the Ar, Ar-0.4% O_2_, and Ar-0.4% N_2_ in ambient air with an output voltage of 3.16 kV and an Ar flow rate of 3l/min with the energy of 0.2 J/sec are shown in Figure S1. The emission lines are identified as per the reported values^13^,^17^. Through the spectra, we observed the emission lines that resemble NO radicals at 283.37 nm, OH radicals at 309 nm, N_2_ second positive system peaks at 336.01 nm, 357.21 nm, and 379.68 nm, and atomic oxygen (O) at 777.23 nm. Additionally, we observed the emission peaks of Ar lines at 696.44, 706.59, 727.35, 738.46, 750.32, 763.41, 772.46, 794.78, 826.51, 842.44, 852.14, 911.97, and 922.3 nm. Moreover, the species at wavelengths of 336 and 357 nm is defined for the NO β^2^ ∏ or N_2_^3^∏ (hereafter NO/N_2_) because both species have possible optical emissions at these wavelengths^13^,^17^. Further, we compare the emission lines for NO, OH, NO/N_2_, Ar, and O at 283, 309, 337, 763, and 777 nm, respectively, at two places, near the ground electrode (point 1) and at 2 mm from the ground electrode (point 2), as shown in Fig. 2. The results are very interesting in both places (point 1 and point 2), where the intensity of the reactive species decreased as the distance increased. Moreover, for the Ar gas plasma at point 1, the Ar emission lines at 763 nm are of very high intensity followed by the OH emission lines at 309 nm. The presence of O (ground state or excited state) has a significant role in plasma medicine due to its reactivity with the biological samples^13^,^18^.

When we add O_2_ to a certain amount of Ar plasma, the excited Ar species can be consumed in the plasma downstream by the O_2_ through metastable neutral collisions or Penning ionization processes. Therefore, in order to determine the change in radical chemistry with the addition of O_2_ in Ar plasma, we observed the change in the emission lines of the NO, OH, NO/N_2_, Ar, and O reactive species at both point 1 and point 2. At point 1, we observed that the NO, OH, NO/N_2_, and O emission lines intensity decreases, while the peaks at 763 nm of the Ar emission lines did not change with the increase in O_2_ content. On the other hand, at point 2, the emission line of NO at 283 nm was not observed for Ar plasma or for Ar-O_2_ plasma, while for the NO/N_2_ peak at 337 nm, the emission line decreases in Ar at point 2 compared with at point 1. However, for the Ar-O_2_ gas plasma mixtures (0.2%, 0.3%, and 0.4% O_2_ addition), the peak at 337 nm was not observed. On the other hand, the emission peak of Ar at 763 nm decreases with the addition of O_2_ content. Moreover, the peak of OH at 309 nm decreases with the addition of O_2_ content compared to Ar plasma; further increases in O_2_ content did not significantly affect the peak of OH radicals. Surprisingly though, the intensity of O at 777 nm did not change significantly with the increase in O_2_ content.

Similarly, for the N_2_ addition, we observed a change in the chemistry of radicals at point 1. The Ar peak at 763 nm did not change with the increase in the N_2_ content, similar to the O_2_ addition for all studied systems. On the other hand, the OH and O peaks decreased with the addition of N_2_ content compared with Ar plasma, but among the O_2_ mixtures, no change in intensity was observed. However, the peaks of NO/N_2_ increased with the increase in N_2_ content compared to Ar plasma. At point 2, no NO peak at 283 nm was observed for Ar plasma, but it was present in the case of the Ar-N_2_ plasma at all mixtures. Similar to point 1, the peak for NO/N_2_ increased as the N_2_ content increases. The change in the OH peak did not vary significantly with the increase in N_2_ content. Moreover, the peaks at 763 and 777 nm decreased as the N_2_ content increased, similar to that for Ar and O, respectively, as shown in Fig. 2.

### Change of physical and chemical composition of DI water after treatment with mixed gas plasmas

**Figure 3:**
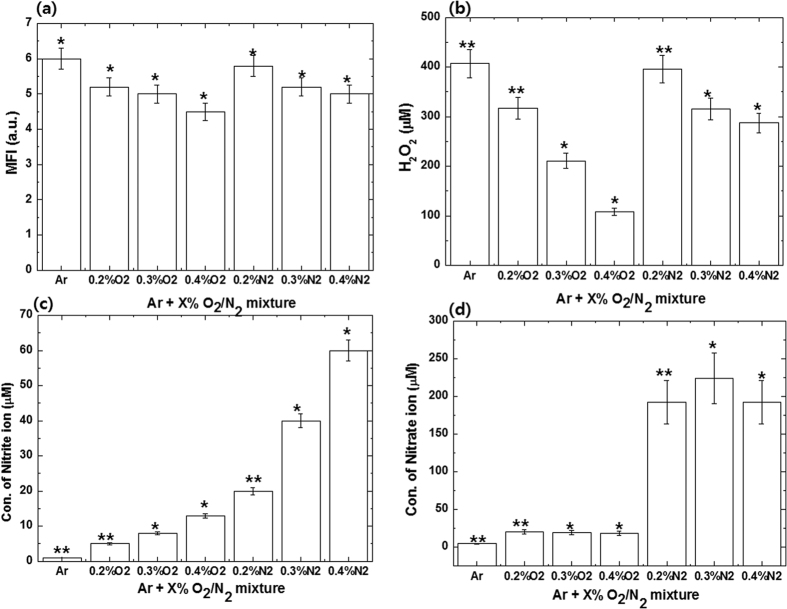
Generation of reactive species (**a**) OH radicals; (**b**) H_2_O_2_; (**c**) Nitrite ions; (**d**) Nitrate ions, after DBD jet treatment for 4 min using feeding gases such as Ar, Ar-O_2_, and Ar-N_2_ at different ratios in DI water.

As can be seen from the above study, different types of RONS were produced by CAP in the gas phase by changing the feeding gas mixtures. Therefore, in order to determine the types of RONS produced by the CAP in water, different feeding gases such as the Ar, Ar-O_2_, and Ar-N_2_ mixtures were used at different ratios for 4 min treatments, as shown in Fig. 3. In the DI water, we studied the hydroxyl (OH) radicals, hydrogen peroxide (H_2_O_2_), nitrite (NO_2_^−^) ions, and nitrate (NO_3_^−^) ions. However, as the addition of O_2_ and N_2_ increases, the intensity of RONS increases or decreases depending on the reactive species. Similar to this behavior, we observed that the RONS concentration varies with the addition of the O_2_ and N_2_ at different ratios.

First, we observed the OH radicals and found that after 4 min treatment in the Ar and Ar-O_2_ plasma, the fluorescence intensity generated due to the reaction of terephthalic acid (TA) with OH (as described in our earlier work)^3^,^15^,^16^, decreases with the addition of O_2_ content. Similarly, for Ar-N_2_, the intensity decreases with the addition of N_2_ content from 0.2% to 0.4%. Moreover, a comparison shows that both intensities decrease more with the addition of O_2_ content than with that of N_2_ content. The same pattern will follow for the H_2_O_2_, in which Ar plasma can produce the highest H_2_O_2_ (407 μM) for the 4 min treatment, while it decreases as the contents of O_2_ (108 μM) and N_2_ (287 μM) increase. We then checked the NO_2_^−^ ions and NO_3_^−^ ions in all mixtures for 4 min treatments in DI water. From Fig. 3, we observed that the concentration of NO_2_^−^ ions increased with increases of O_2_ content. For Ar plasma, the concentration of NO_2_^−^ ions is considerably less at around 2 μM, but for 0.4% of O_2_ it increases to 13 μM. However, we observed similar behavior for the addition of N_2_ content, but the increase of NO_2_^−^ ions for the N_2_ content is considerably more than for the O_2_ content. The concentration of NO_2_^−^ ions for 0.4% of N_2_ content was 60 μM. On the other hand, the changes in the concentration of the NO_3_^−^ ions significantly differed with the increase in the contents of O_2_ and N_2_ in Ar plasma. While the NO_3_^−^ ions concentration increases with a small addition of O_2_ content, it decreases slightly if the O_2_ content increases. Moreover, similar trends were observed for the effect of the addition of N_2_ content on the concentration of NO_3_^−^ ions, where the concentration increases for 0.3% but decreases for the 0.4% N_2_ addition. However, if we closely examine the concentration values of the NO_3_^−^ ions compared with those of the NO_2_^−^ ions for N_2_ content addition, the difference is significant. The NO_2_^−^ ions concentration at 0.4% N_2_ was 60 μM, while the concentration of NO_3_^−^ ions at the same mixture ratio was 180 μM. However, the NO_2_^−^ ions concentration at 0.4% O_2_ was 13 μM, while the concentration of NO_3_^−^ ions at the same mixture ratio was 20 μM. Similar trends of RONS were observed for 1 and 2 min treatment of Ar, Ar-O_2_, and Ar-N_2_ with all additions of plasma mixture ratios (data not shown). On the other hand, the change in temperature is not significant after the treatment at all mixture ratios of plasma compared to the control, as shown in Figure S2. We observed similar behavior of RONS for 1 min and 2 min treatment in all mixtures at all ratios (Ar, Ar-O_2_, and Ar-N_2_) (data not shown), while the pH change is very prompt for the addition of N_2_ content compared to the addition of O_2_ content. After 4 min Ar CAP treatment, the pH changes from 6.2 to 5.9, while for the 0.2% addition of N_2_ content, the pH decreases to 4.2; however, with further addition of N_2_ content it does not change significantly. However, for the 0.2% of O_2_ content, the pH decreases slightly to 5.2 compared to Ar plasma. We observed similar behavior of pH for 1 min and 2 min treatments in all mixtures at all ratios (Ar, Ar-O_2_, and Ar-N_2_) (data not shown). We then tested our model proteins, Hb and Mb, in all mixture ratios for 1, 2, and 4 min treatments.

### Circular dichroism (CD) and fluorescence analysis for Hb and Mb after Ar DBD plasma jet treatment

**Figure 4:**
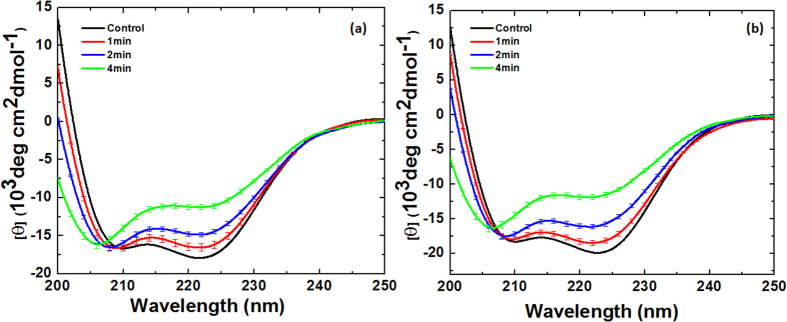
CD spectra of (**a**) Hb and (**b**) Mb at different time interval treatments.

**Figure 5:**
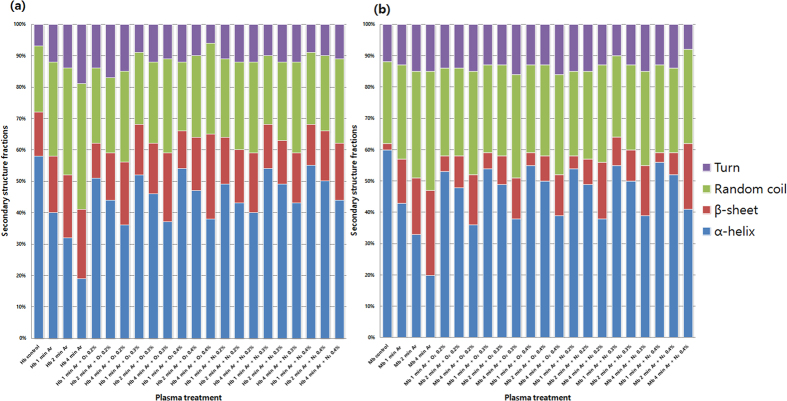
Secondary structure changes in (**a**) Hb and (**b**) Mb at different time intervals and at different mixed ratios.

To understand the degree of modification using Ar DBD jet plasma on Hb and Mb, we performed circular dichroism (CD) analysis. As seen in Fig. 4a,b, the far-UV CD spectra of Hb and Mb indicate changes in the secondary structure due to the increased treatment time. The two strongly pronounced minima at ~210 and ~222 nm for Hb and Mb, respectively, indicate the α-helix conformation of the polypeptide chain. The results in Figs 4a and 5a clearly reveal that for the control Hb, 58% of α-helical, 14% of β-sheet, and 21% random coil are observed. On the other hand, for 1 min treatment, the α-helical decreases to 40% and β-sheet increases to 18%. Similar to the 2 and 4 min treatments, the α-helical decreases to 32% and 19%, while the β-sheet increases to 20% and 22%, respectively. On the other hand, for the Mb treatment, we observed the α-helical, β-sheet, and random coil to be 60%, 2%, and 26%, respectively, as shown in Figs 4b and 5b. For the 1, 2, and 4 min treatments, the α-helical decreases to 43%, 33%, and 20%, respectively, while the β-sheet increases to 14%, 18%, and 27%, respectively. This shows that for both Hb and Mb, the α-helical decreases and β-sheet increases, as illustrated in Fig. 5.

We also studied the fluorescence analysis to determine the heme degradation using the previously reported method^15^,^19^,^20^,^21^, providing the excitation at 321 nm and emission at 460 nm as shown in Figure S3, for Hb and Mb, respectively. The heme degradation can be identified by the presence of fluorescence intensity at 460 nm^15^. We studied the fluorescence intensity for the Ar DBD jet for different time intervals, and observed that as the treatment increases, the fluorescence intensity also increases, implying that the degradation of heme increases. Similar to that for the Mb, the fluorescence intensity increases as the treatment time increases.

### CD and fluorescence analysis for Hemoglobin after Ar-O_2_ and Ar-N_2_ DBD plasma jet treatment

**Figure 6:**
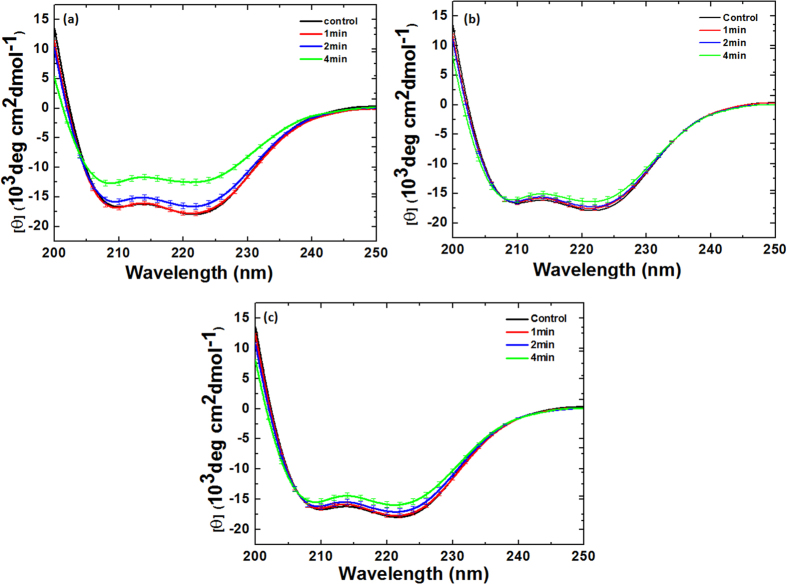
CD spectra of Hb treatment at different time intervals and at different Ar-O_2_ ratios (**a**) 0.2% O_2_; (**b**) 0.3% O_2_, and (**c**) 0.4% O_2_.

**Figure 7:**
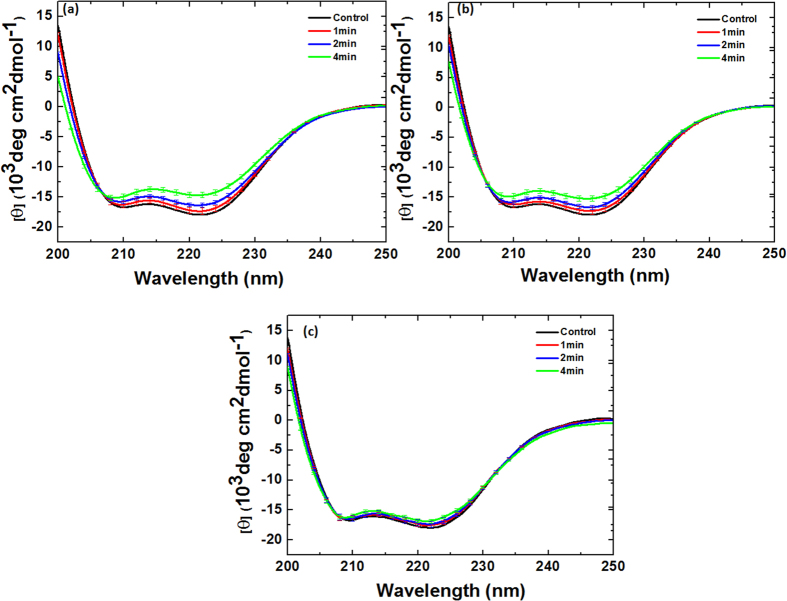
CD spectra of Hb treatment at different time intervals and at different Ar-N_2_ ratios (**a**) 0.2% N_2_; (**b**) 0.3% N_2_, and (**c**) 0.4% N_2_.

The CD analysis above shows that the structure of Hb changes as the treatment time increases. In order to check the effect of the mixture ratios on the structure of Hb, we added 0.2%, 0.3%, and 0.4% O_2_ content and treated for 1, 2, and 4 min for each ratio. The structure of Hb shows somewhat different behavior when the O_2_ content is added to Ar plasma at different ratios of 0.2%, 0.3%, and 0.4% and treated for 1, 2, and 4 min for each ratio compared with Ar plasma treatment, as shown in Figs 5a and 6. For the treatment of Hb at 0.2%, 0.3%, and 0.4% O_2_ content for 1 min, the change in α-helical was 51%, 52%, and 54%, respectively. For 0.2%, 0.3%, and 0.4% O_2_ content at 2 min, the change in α-helical was 44%, 46%, and 47%, respectively. For the 4 min treatment at 0.2%, 0.3%, and 0.4% O_2_ content, the change in α-helical was 36%, 37%, and 38%, respectively. For the treatment of Hb for 1, 2, and 4 min with 0.2% N_2_ content, the change in α-helical was 49%, 43%, and 40%, respectively, which is quite close to the control (58% α-helical), as shown in Figs 5 and 7. For 0.3% N_2_ content, the change in α-helical was 54%, 49%, and 43% for 1, 2, and 4 min, respectively. For the 0.4% N_2_ content treatment for 1, 2, and 4 min, the change in α-helical was 55%, 50%, and 44%, respectively. This shows that the change in α-helical is very similar for the 0.3% and 0.4% N_2_ additions.

In order to understand the effect of mixed gases such as O_2_ and N_2_ with Ar DBD jet on the heme degradation of hemoglobin protein, we used fluorescence spectroscopy, the results of which are shown in Figures S4 and S5. We added 0.2%, 0.3%, and 0.4% O_2_ content and treated for 1, 2, and 4 min for each ratio to check the heme degradation. We provided the excitation at 321 nm and studied the emission at 460 nm, as shown in Figures S4 and S5. This shows that for the 1 and 2 min treatments of Hb at 0.2%, 0.3%, and 0.4% O_2_ content, the change in α-helical is insignificant compared with the control, while for 4 min treatment at all ratios of O_2_ content the change in α-helical is significant. This shows that degradation increases considerably more for the 4 min treatment than for the 1 and 2 min treatments for any ratio of O_2_ content. The fluorescence intensity value decreases at 4 min treatment with the increase in O_2_ content, as shown in Figure S4. Similarly, for the Ar-N_2_ mixture, the difference in fluorescence intensity is considerably less for the 1 min and 2 min treatments at 0.3% and 0.4% additions of N_2_ content, as shown in Figure S5, while the difference in fluorescence intensity is significant for the 2 min treatment at 0.2% addition of N_2_ content. In addition, at the 4 min treatment with increase in N_2_ content, the fluorescence intensity decreases, showing very similar behavior to that of the Ar-O_2_ plasma.

### CD and fluorescence analysis for Myoglobin after Ar-O_2_ and Ar-N_2_ DBD plasma jet treatment

**Figure 8:**
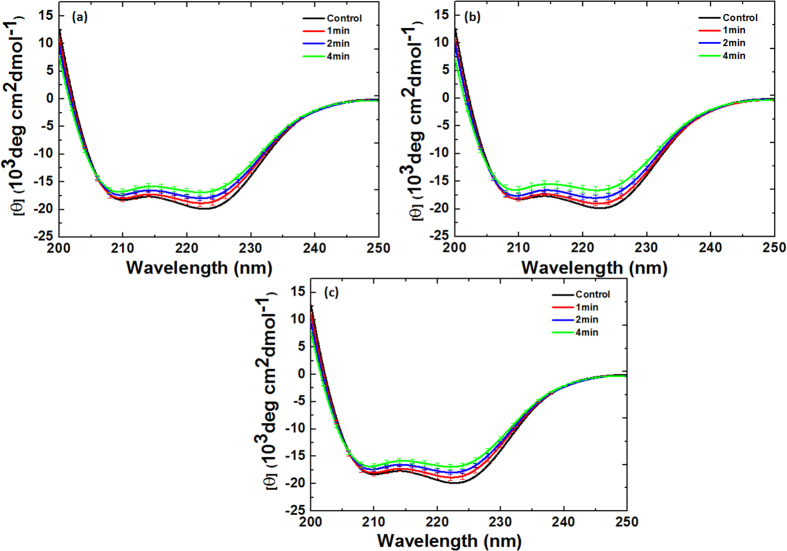
CD spectra of Mb treatment at different time intervals and at different Ar-O_2_ ratios (**a**) 0.2% O_2_; (**b**) 0.3% O_2_, and (**c**) 0.4% O_2_.

The CD analysis above shows that the structure of Mb changes as the treatment time increases. To determine the effect of the mixture ratios on the structure of Mb, we added 0.2%, 0.3%, and 0.4% O_2_ content and treated for 1, 2, and 4 min for each ratio, as shown in Figs 5b and 8. With the treatment of Mb for 1, 2, and 4 min with 0.2% O_2_ content, the change in α-helical was 53%, 48%, and 36%, respectively. For the 0.3% O_2_ content, the change in α-helical was 54%, 49%, and 38% for the 1, 2, and 4 min treatments, respectively, while for the 0.4% O_2_ content treatment at 1, 2, and 4 min, the change in α-helical was 55%, 50%, and 39%, respectively.

**Figure 9:**
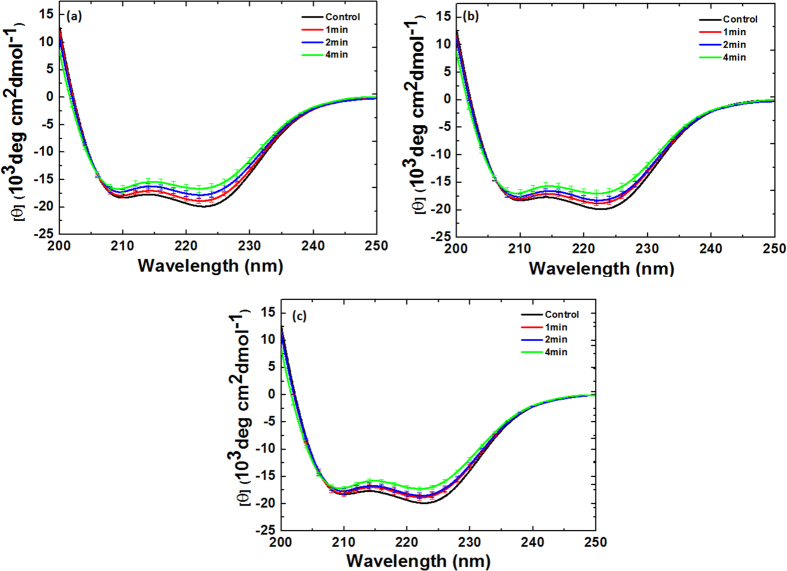
CD spectra of Mb treatment at different time intervals and at different Ar-N_2_ ratios (**a**) 0.2% N_2_; (**b**) 0.3% N_2_, and (**c**) 0.4% N_2_.

On the other hand, the structure of Mb shows somewhat different behavior when the N_2_ content is added to Ar plasma at different ratios of 0.2%, 0.3%, and 0.4% and treated for 1, 2, and 4 min for each ratio, as shown in Figs 5b and 9. With the treatment of Mb for 1, 2, and 4 min with 0.2% N_2_ content, the change in α-helical was 54%, 49%, and 38%, respectively. For the 0.3% N_2_ content, the change in α-helical was 55%, 50%, and 39% for the 1, 2, and 4 min treatments, respectively. On the other hand, for the 0.4% N_2_ content treatment at 1, 2, and 4 min, the α-helical change was 56%, 52%, and 41%, respectively. This shows that the change in α-helical is very similar for all ratios of O_2_ addition; while the difference is diminutive, the trends are quite similar, whereby α-helical decreases and β-sheet increases with increase in treatment time. This shows that the change in α-helical is very similar for all ratios of O_2_ and N_2_ addition; the difference is diminutive but the trend is quite similar, whereby α-helical decreases and β-sheet increases with increase in treatment time.

We then checked the fluorescence analysis to understand the effect of mixed gases such as O_2_ and N_2_ with Ar DBD jet on the heme degradation of myoglobin protein, as shown in Figures S6 and S7. We added 0.2%, 0.3%, and 0.4% O_2_ content and treated for 1, 2, and 4 min for each ratio to determine the heme degradation for Mb. We provided the excitation at 321 nm and studied the emission at 460 nm as shown in Figures S6 and S7. This demonstrates that the 1 and 2 min treatments of Hb at 0.2%, 0.3%, and 0.4% O_2_ content do not show significant change compared with the control, while for 4 min treatment at all ratios of O_2_ content, the change is very pronounced. This shows that the degradation increases considerably for the 4 min treatment compared to 1 and 2 min for any ratio of O_2_ content. The fluorescence intensity value decreases at 4 min treatment of Mb with increase in O_2_ content, as shown in Figure S6. Similarly, for the Ar-N_2_ mixture, the difference in fluorescence intensity is almost similar to that for the 1 min and 2 min treatments at the 0.2%, 0.3%, and 0.4% additions of N_2_ content. In addition, at 4 min treatment with increase in N_2_ content, the fluorescence intensity did not decrease significantly, as shown for the Hb case. The above experiments of the CD and fluorescence analysis reveal major structural changes for the 4 min treatment and minimum change for the 0.4% addition of O_2_ or N_2_ to the Ar DBD jet. Therefore, for further analysis, we used Fourier transform infrared (FTIR) spectroscopy.

### Fourier transform infrared (FTIR) spectroscopy analysis of Hb and Mb treatment

The FTIR technique is a valuable tool in the examination of protein conformation. The region of the Amide I band has most frequently been used to evaluate the secondary structure of proteins. The stretching vibration of carbon and oxygen of the peptide carbonyl functional groups contribute to the amide I band in the range of 1700 and 1600 cm^−1^ 22,23. In this work, we studied the peaks at 1650 and 1630 cm^−1^ using the attenuated total reflectance (ATR) sampling technique, as illustrated in Figure S8. To avoid the interference of water in treated and non-treated samples, we dyed the samples at room temperature in a dry place, and then measured the samples using FTIR spectroscopy. The FTIR peaks at 1650 cm^−1^ belong to α-helical and the peak at 1630 cm^−1^ represents the extended chain^24^,^25^. We also summarized our Hb FTIR data in terms of these above mentioned peaks for the 0.4% addition of O_2_ or N_2_ to the Ar DBD jet for 1, 2, and 4 min treatments. We also verified the peaks in the Ar DBD jet for 1, 2, and 4 min treatments. For the Ar DBD jet treatment of Hb, we observed that the α-helical peak at 1650 cm^−1^ decreases as the treatment time increases, whereas the difference in peak intensity is higher for the 4 min treatment than for the 1 and 2 min treatments. On the other hand, the 0.4% O_2_ content plasma treatment reveals that the intensity of the α-helical peak decreases as the treatment time increases, but it decreases less than with the Ar plasma. Similar results were observed for the 0.4% N_2_ content plasma treatment of Hb protein, in which the intensity of the α-helical peak decreases as the treatment time increases, but the intensity is more than that for the 0.4% O_2_ plasma as shown in Figure S8. Similar trends were seen for the peak at 1630 cm^−1^ which represents the extended chain of Hb protein. Ar plasma also has a strong effect on the extended chain. The intensity at 1630 cm^−1^ decreases as the treatment time increases for the Ar-O_2_ and Ar-N_2_ plasma, but the decrease in intensity is considerably less than that of the Ar plasma.

A similar pattern of behavior was observed for the Mb protein, in which the intensity of the peaks at 1650 and 1630 cm^−1^ decrease as the treatment time increases. Additionally, for the 0.4% O_2_ and 0.4% N_2_ mixture gases plasma, the same trend is observed, but the difference is less than in the Ar-DBD jet plasma.

### Molecular dynamics simulation of Hb and Mb in the presence of H_2_O_2_

To understand the influence of hydrogen peroxide (H_2_O_2_) on the Hb and Mb structures, we used MD simulations to analyze the structural change of these paralogs. We prepared two types of MD simulation systems: with the protein solvated in pure water and with 10% H_2_O_2._ By measuring the root-mean-square atomic positional deviation (RMSD) values, we were able to compare the structural stabilities of Myoglobin and Hemoglobin in each environment and identify the influence of H_2_O_2_ on these proteins.

**Figure 10:**
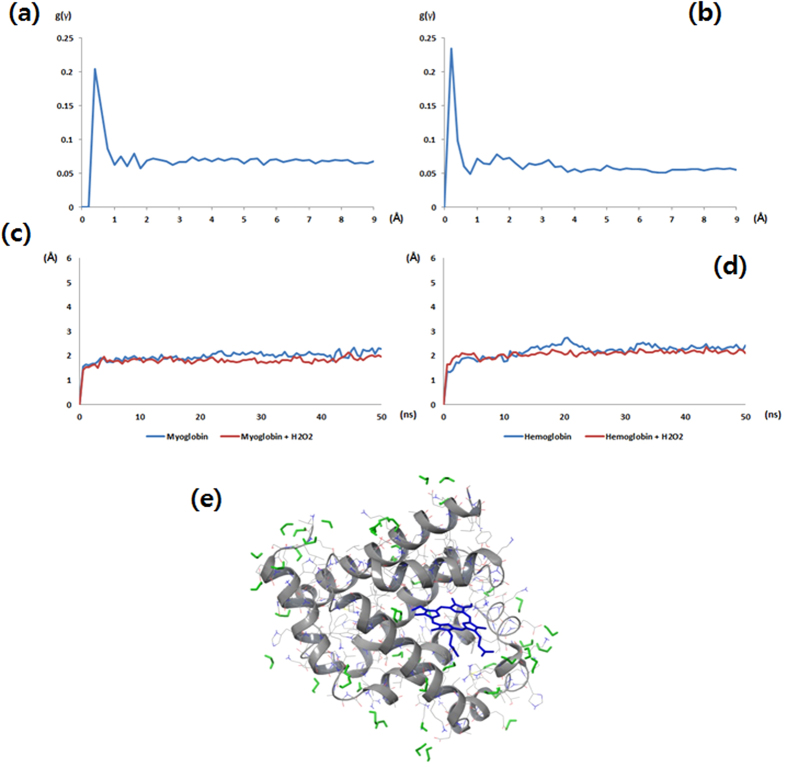
Molecular dynamics simulation results of Hb and Mb, (**a**) radial distribution function between the Mb and H_2_O_2_; (**b**) radial distribution function between the Hb and H_2_O_2_; (**c**) RMSD plots of the Mb in the pure solvent and 10% H_2_O_2_ containing solvent; (**d**) RMSD plots of the Hb in the pure solvent and 10% H_2_O_2_ containing solvent; (**e**) snapshot of Mb with surface H_2_O_2_ (gray ribbon: myoglobin, green ribbon: H_2_O_2_ and blue ribbon: heme).

We first calculated the radial distribution function (RDF) between the proteins and H_2_O_2_, which gives the probability of finding a H_2_O_2_ molecule at a distance γ from the proteins. The RDF values from the Hb and Mb simulations are shown in Fig. 10a,b, respectively. The H_2_O_2_ molecules were initially uniformly distributed in solutions, but they condensed within 1Å of the protein surfaces in a relatively short period of time. Subsequently, we measured the RMSD values (Fig. 10c,d). The H_2_O_2_ molecules interacted with the surface of Mb (Fig. 10e). H_2_O_2_ is a strong oxidizing agent and its accessibility to the proteins was identified in the RDF values. However, MD simulations (usually performed with force fields) cannot properly describe chemical reactions such as oxidation. The measured RMSD values indicate that the presence of H_2_O_2_ does not affect the stability of the Hb and Mb structures in the MD simulations.

**Figure 11:**
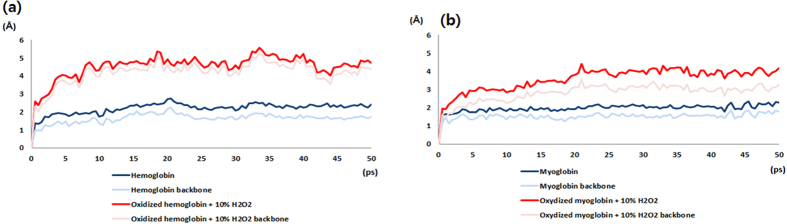
RMSD changes of Hb and Mb in the oxidation state with or without 10% H_2_O_2_ containing solvent (**a**) Hb and (**b**) Mb.

Therefore, in order to determine how oxidation influences the two globin family proteins, we manually changed them to oxidation states and performed MD simulations. Based on the prior simulation results, the side chains which had interactions with H_2_O_2_ were selected. Among them, only Cys, Met, Trp, Phe, Tyr, His, Arg, Lys, Pro, and Thr were changed to oxidation states because the oxidation states were determined experimentally only for those residues^26^. Using the oxidized structures, we performed further MD simulations and measured the RMSD values (Fig. 11). The RMSD of Hb and Mb in pure solvent reached a stable value after 500 ps and remained at about 2 Å. However, the proteins in 10% H_2_O_2_ containing solvent could not enter an equilibrium state and the RMSD values continued to fluctuate during 50 ns simulation. The averages of the RMSD values were 4.52 Å and 3.57 Å in the simulations of Hb and Mb, respectively. Not only the side chains but also the protein backbones fluctuated considerably more than in pure solvent. From these results, it can be inferred that the protein oxidation by H_2_O_2_ destabilizes the Hb and Mb structures.

## Discussion

The mixed gas plasmas can create different types of chemistries in the gas phase compared to the solution phase. To understand the reason for this, we studied the OES spectra at two points (point 1 and point 2) of the three types of feeding gas plasmas: (1) Ar gas plasma, (2) Ar-O_2_ [0.2%, 0.3%, and 0.4% O_2_ content added to Ar gas], and (3) Ar-N_2_ [0.2%, 0.3%, and 0.4% N_2_ content added to Ar gas]. Figure 2 shows that the Ar DBD jet plasma at point 1 has NO, OH, NO/N_2_, Ar, and O peaks, but at point 2, it has OH, NO/N_2_, Ar, and O peaks. This data reveals that as the distance increases, the intensity of the peaks decreases and the NO peak disappears due to the continuous reaction among the radicals. The addition of O_2_ content results in a decrease of all peaks except the Ar peak at point 1, while at point 2, all peaks decrease as the O_2_ content increases, except for the O peak. The addition of N_2_ content in the Ar DBD jet plasma also changes the gas chemistry. At point 1 for N_2_ addition, the Ar lines remain stable for all added mixtures, while for the other reactive species, the NO, OH, and O peaks decrease as the N_2_ content increases, except for NO/N_2_. The peak for NO/N_2_ increases as the N_2_ content increases; this is due to the increased generation of the N_2_ second positive system to the N_2_ inflow. At point 2, for the Ar DBD jet plasma, the NO peak disappears, while for the N_2_ content it appears at point 2, and the intensity does not change as the N_2_ content increases. This shows that the addition of N_2_ content increases the generation of NO in the gas phase with the addition of a small amount of N_2_ gas, but does not change if further N_2_ is added. Similar to point 1, at point 2 the N_2_/NO intensity also increases as the N_2_ content increases.

If we compare the Ar peak at 763 nm for the Ar-O_2_ and Ar-N_2_ admixtures, we find that the change in Ar-N_2_ is very slight as the N_2_ content increases, while the change is significant when the O_2_ content increases. The intensity of all species decreases with the addition of O_2_ content because, with the addition of O_2_ content, the fraction of the absorbed power is converted into rotational and vibrational excitation of the molecules. Therefore, O_2_ or O does not readily release its energy upon surface impact and it can be pumped away as a hot exhaust gas^27^. Additionally, O_2_ is an electronegative gas, so it decreases the number of electrons by attachment, resulting in the formation of O^−^ and O_2_^−^ ions^28^. Moreover, many other possible reactions can occur as shown in equations 1 to 9 (Table 1).

For the for Ar-N_2_ admixture plasma, the N atom density reaches its maximum with a small addition of N_2_ content at fixed power. Therefore, any further addition of N_2_ content does not increase the N production. Hence, while the NO content did not increase further with the addition of N_2_ content, the NO/ N_2_ content increases due to the continued collision of Ar* to N_2_ gas.

The concentration of reactive species in DI water depends on the RONS generated during and after treatment. We measured the OH, H_2_O_2_, NO_2_^−^, and NO_3_^−^. As seen above for the RONS analysis, for the Ar DBD jet plasma, the concentrations of OH and H_2_O_2_ were high and they increase with the increase in the time of treatment, although the concentrations of NO_2_^−^ and NO_3_^−^ ions are very low. On the other hand, the concentrations of OH and H_2_O_2_ decrease for admixtures of O_2_ and N_2_ content in Ar DBD jet plasma. For the NO_2_^−^ and NO_3_^−^ ions, the concentration is very high for the N_2_ addition compared to the O_2_ addition and Ar plasma. The concentration of NO_2_^−^ increases as the percentage of O_2_ and N_2_ content increases for the 4 min treatment. The NO_3_^−^ ions increased suddenly with the addition of 0.2% N_2_ or O_2_ content and they do not change with further addition of N_2_ and O_2_ content. This might be due to the rapid reaction between the excited N_2_ and O_2_ molecules, and as described above, at fixed power, further generation of excited N_2_ and O_2_ is not possible; therefore, they remain constant. When we checked the pH of the added mixtures, we found that the decrease in pH with the increase in O_2_ content is small, but the decrease is very high for the N_2_ content. While the pH decreases with only a small amount of N_2_ content (0.2%), a further increase in N_2_ content does not significantly change the pH. Hence, in the above two results, the generation of NO_3_^−^ and the decrease of pH with 0.2% N_2_ content are well correlated. The following reactions 10 and 11 are responsible for the decrease in the pH of water


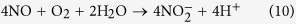



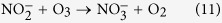


Therefore, in order to determine the reactive oxygen species (ROS) and reactive nitrogen species (RNS) on proteins, we tested Hb and Mb using CD and fluorescence spectroscopy. The CD data reveal that as the treatment time increases, the percentage of α-helical decreases and the percentage of β-sheet increases; this trend is also the same for admixtures. This also correlates with previous work by other authors, in which a similar trend after plasma treatment was also observed^32^,^33^. However, the fluorescence analysis shows the degradation of the heme group in protein; the hemes are non-fluorescent, but during the heme degradation, the fluorescent porphyrin degradation product yield increased^15^,^19^,^20^,^21^. Comparing the percentage of α-helical decreases between the O_2_ and N_2_ admixtures at all mixture ratios (O.2%, 0.3%, and 0.4%) and at all treatment times (1, 2, and 4 min), we found that the α-helical decreases more for O_2_ admixtures than for N_2_ admixtures. Similar trends were also observed for the FTIR data, where the greatest percentage of α-helical decrease was for Ar, followed by Ar-O_2_ mixture and the least percentage of α-helical decrease was for the Ar-N_2_ mixtures. The values of ROS (OH, H_2_O_2_) for Ar plasma are higher than those for other admixtures (Ar-O_2_ and Ar-N_2_); therefore, more structural changes are observed in the case of Ar plasma. Additionally, for O_2_ admixtures, the role of O can be important for the structural changes of proteins, as the concentrations of OH, H_2_O_2_, NO_2_^−^, and NO_3_^−^ are less for the O_2_ admixture than for the N_2_ admixture, but the structural changes for O_2_ admixtures are greater than for those for the N_2_ admixtures. Moreover, a significantly greater number of short lifetime radical species such as superoxide (O_2_^•−^), atomic oxygen (O), peroxynitrite (ONOO^−^), etc. can be produced by Ar-O_2_ than by Ar-N_2_; this can play an important role in the structural changes of Hb and Mb. The previous results by Bogaert’s group^34^ show that the O and OH radicals play an important role in the H atom abstraction from the fatty acid and they change the lipid composition of the skin. Hence, both radicals can be responsible for the structural modification of both proteins during Ar-O_2_ treatment.

We also observed the heme degradation of the fluorescence intensity at 460 nm. We found that in all the admixtures (O_2_ and N_2_) and at all ratios, the fluorescence intensity increases as the time of treatment increases. However, the fluorescence intensity decreases as the admixture ratios increase, while no significant difference in intensity was observed for the 1 and 2 min treatments for both proteins (Hb and Mb). In the previous reports, the researchers observed that the OH/H_2_O_2_ radicals are mainly responsible for the heme degradation^19^,^20^,^21^. Therefore, for the Ar gas plasma, the OH and H_2_O_2_ concentrations increase as the treatment time increases, so that the degradation also increases. Similarly, for the O_2_ and N_2_ admixtures, the generation of OH and H_2_O_2_ increases as the treatment time increases, but the amount of increase is less than that for Ar plasma. Therefore, the heme degradation was also less for the admixtures than for Ar plasma, as shown in Figures S3–S7.

The experimental data obtained by our group and by other groups on the RONS demonstrated that H_2_O_2_ is one of the main and stable species that can result in structural changes of biomolecules due to its oxidation process and it can enhance anticancer therapy^9^,^10^,^12^,^35^ Therefore, for better understanding, we verified the molecular dynamic simulation using 10% H_2_O_2_ with Hb and Mb. We measured the RMSD values, which indicate that the presence of H_2_O_2_ does not affect the stability of Hb and Mb structures in MD simulations. Therefore, to understand how oxidation influences the Hb and Mb proteins, we manually changed them to oxidation states and performed MD simulations. Based on the prior simulation results, the side chains which interacted with H_2_O_2_ were selected. The RMSD of Hb and Mb in pure solvent reached stable values after 50 ns and remained at about 2 Å. However, the proteins in the 10% H_2_O_2_ containing solvent could not enter an equilibrium state and the RMSD values continued to fluctuate during 50 ns of simulation. Additionally, we found that the protein backbones fluctuated considerably more than in pure solvent. Therefore, these results reveal that the protein oxidation by H_2_O_2_ destabilizes Hb and Mb structures.

Finally, we conclude that Ar gas plasma generates more ROS than the admixtures (when the solution is treated from a fixed position without changing the plasma characteristics such as flow rate of gas, treatment time, and voltage), which directly affects the structural changes of the Mb and Hb proteins. However, the addition of a small amount of O_2_ decreases the plasma density that affects the ROS concentration in the solution, which is related to the fewer structural changes in the Ar-O_2_ plasma mixture than in the Ar plasma. However, while the addition of N_2_ increases the RNS concentration in plasma, resulting in a high concentration of NO_2_^−^ and NO_3_^−^ in the solution, it does not reveal more structural changes than other admixtures (Ar and Ar-O_2_). Hence, RNS plays a minor role in the structural changes. The increase of RNS concentration has a more important role in decreasing the pH of the solution, and these pH changes affect the structure of proteins and help the radicals to react rapidly. Nevertheless, compared to the N_2_ admixtures, the O_2_ admixture affects major changes in the structures of Mb and Hb, as seen in the CD and FTIR analyses. This shows that other short lifetime radical species (such as O_2_^•−^, O, ONOO^−^, etc.) generated through O_2_ admixtures play more prominent roles than other long lifetime radicals for structural changes of protein, although further details need to be explored in future work.

## Experiment Section

### Materials

The Hemoglobin and Myoglobin proteins were supplied by Aldrich Chemical Co. (USA). All chemicals and reagents were used without any further purification. The H_2_O_2_ was measured using titanyl ion, and NO was detected using 4-amino-5-methylamino-2′,7′-difluorofluo rescein (DAF-FM)^36^. OH was measured using terephthalic acid (20 mM), as per the procedure given in the earlier research work^3^,^11^,^36^. NO_2_^−^ was measured using the Griess reagent supplied by Aldrich Chemical Co. (USA)^36^. NO_3_^−^ was measured using the Acorn Series ION 6 meter (pH/mV/°C Meter), nitrate electrode, from Oakton Instruments, USA^36^.

### Fluorescence spectroscopy

The fluorescence spectroscopy instrument used for measuring the fluorescence intensity in the present investigation is similar to that used in our earlier works^37^,^38^. Steady-state fluorescence measurements were carried out using a Perkin Elmer LS 55 fluorescence spectrometer. The excitation wavelength was fixed at 321 nm to obtain the contribution of the degradation of the heme group from the overall fluorescence emission. The slit widths of the excitation and emissions were both set at 10 nm. The concentration for this experiment is 1 mg/ml for both proteins (Hb and Mb).

### Circular dichroism spectroscopy

CD spectroscopic studies^37^,^38^,^39^,^40^ were performed using a J-815 spectrophotometer (Jasco, Japan) equipped with a Peltier system for controlling the temperature. (1S)-(+)−10-camphorsulfonic acid (Aldrich, Milwaukee, WI) was utilized for CD calibrations, exhibiting a molar extinction coefficient of 34.5 M/cm at 285 nm, and molar ellipticity (θ) of 2.36 M/cm at 295 nm. The samples were pre-equilibrated at the desired temperature for 15 min and the scan speed was fixed for adaptive sampling (error F 0.01) with a response time of 1 s and bandwidth of 1 nm. The secondary structures of Hb and Mb were monitored using a 1.0 mm path length cuvette. The concentrations for the secondary structures of Hb and Mb were 0.2 mg/ml, each spectrum being an average of six spectra. Each sample spectrum was obtained by subtracting appropriate blank media without Hb and Mb from the experimental proteins spectrum. The percentages of secondary structures were then calculated using Yang’s method^41^.

### Fourier transform infrared spectroscopy (FTIR)

The FTIR spectroscopy analyses were analyzed using the Thermo scientific NICOLET iS10-ATR spectrophotometer with a deuterated triglycine sulfate (DTGS) detector. The proteins in solid state were placed in a horizontal ATR accessory with a zinc selenide prism. All spectra were acquired using four scans with a resolution of 4 cm^−1^ at ambient temperature (25 °C) and repeated four times. To avoid the treatment difference for FT-IR samples and other measurements, we prepared the samples in DI water. The concentrations of Hb and Mb were both 4 mg/ml. After the treatment, the control samples (without plasma treatment) and plasma treated samples at all conditions were dried using dry air; the dry samples were then collected and the measurements were performed.

### Molecular dynamics simulations

Crystal structures of the human myoglobin and hemoglobin were obtained from the (RCSB) protein data bank website (http://www.rcsb.org; the PDB IDs were 1MBN and 1A3N). These structures were treated with the Protein Preparation Wizard of the Schrödinger suite for molecular dynamics (MD) simulations. All water and heat molecules were eliminated and hydrogens were added and minimized using IMPACT 6.6^42^.

To create the oxidation states of myoglobin and hemoglobin, selected residues were manually manipulated to be in the oxidation states and were optimized using the Prime 3.9 side chain prediction module^43^. The oxidized residues were: 7, 12, 14, 16, 24, 31, 33, 34, 36, 37, 39, 42, 45, 46, 47, 48, 50, 51, 55, 56, 62, 63, 64, 67, 70, 77, 78, 79, 81, 82, 87, 88, 93, 95, 96, 97, 98, 100, 102, 106, 113, 116, 119, 120, 123, 133, 138, 140, 145, 146, 147, and 151 in myoglobin and 4, 7, 8, 11, 14, 16, 20, 31, 32, 33, 36, 37, 38, 39, 41, 43, 44, 46, 56, 60, 61, 67, 72, 76, 87, 89, 90, 92, 98, 99, 103, 104, 108, 117, 118, 127, 128, 134, 137, 139, 140, and 141 in hemoglobin. The oxidation states were determined as follows: sulfonic acid for cysteine, sulfoxide for methionine, hydroxytryptophan for tryptophan, dihydroxylphenylalanine for phenylalanine and tyrosine, 2-oxohistidine for histidine, glutamic semialdehyde for arginine and proline, α-aminoadipic semialdehyde for lysine, and 2-amino-3-ketobutyric acid for threonine.

The MD simulations were performed using Desmond 4.4^44^. The simulation systems were prepared using the Desmond system builder. The box shape of the systems was orthorhombic and the size was determined from the 10 Å buffer distance between the solute structures and the simulation box boundary. The systems were solvated with TIP3P model water. Na^+^ or Cl^−^ ions were added to the systems to neutralize the total charge of the system. Hydrogen peroxide (H_2_O_2_) molecules were added to achieve 10% concentration to the oxidation systems. The charges of H_2_O_2_ were determined from the electrostatic potential (ESP) charge fitting with Jaguar 8.7^45^ using the basis set and function of B3LYP/6-31G**. The MD simulations were performed in the (NPT) ensemble with the OPLS2005 all atom force field. A reference temperature of 300K and pressure of 1atm were maintained by the Nose-Hoover thermostat and the Martyna-Tobias-Klein barostat. Before performing the main simulations, a series of minimizations and short MD simulations were performed to relax the model system.

### Dielectric barrier discharge (DBD) plasma jet

The plasma jet consists of two electrodes, a quartz tube, and a gas nipple. The stainless steel needle is placed at the center of the quartz tube as a high voltage electrode. At the surface of the quartz tube, copper tape was used for the ground electrode and the distance between the high voltage electrode and the ground electrode is about 1 mm, as shown in Fig. 1a. The output voltage is 0.7 kV and the output current is 3 mA. The frequency is 16 kHz and the energy is 0.2 J/sec. High purity argon gas was used with and without the addition of different amounts of oxygen and nitrogen (0.2%, 0.3%, and 0.4%) admixtures. The working gas flow rate is 3 l/min and the gas is injected into the plasma jet through the gas nipple. In order to eliminate the air from the chamber, the working gas [Ar, Ar-O_2_ (at all mixed ratios), Ar-N_2_ (at all mixed ratios)] was bled into the chamber for 10 min before the experiment (pumping was not used in this process). The relative humidity was 47% (Anytem HyGRO Co. Serial number: 412CTH) at 25 °C, close to the plasma jet. The distance between ground electrode and surface of treated protein solution is 10 mm (that keep constant for all experiments). The plume length from the end of quartz tube for Ar, Ar-O_2_ (0.4%) and Ar-N_2_ (0.4%) plasmas are 4.9 mm, 2.5 mm and 3.1 mm, respectively. The OES spectra of the CAP emission were recorded using HR4000CG-UV-NIR (Ocean Optics, FL, USA) and optical fiber (QP400-2-SR) with a diameter of 400 mm. The signal was accumulated for 3 min, and the data was analyzed using the Origin 8.0 software package.

### pH and temperature measurement

After exposure of plasma in water, the pH and temperature of the water were measured using a pH meter (Eutech Instruments, Singapore) and Infrared (IR) camera (Fluke Ti100 Series Thermal Imaging Cameras, UK), respectively. All measurements were carried out in triplicate.

### Sample Preparation

Protein stability was analyzed by incubating 1 ml screw-capped vials in deionized water, at 25 °C for 4 h to achieve complete equilibrium. The 1 ml samples were treated in a 4-well plate (SPL Life Sciences Co.) at 6 mm distances from the end of the quartz tube of the DBD plasma jet for different treatment times, and then incubated for 4 h at room temperature. The concentration of proteins after the plasma treatment is determined using the Bradford method^46^. Three samples were treated for each condition to minimize the error.

### Statistical analysis

All values are represented by the mean ± S.D of the indicated number of replicates. Statistical analyses of the data were performed using the student’s t-test to establish the significance between data points, while the significant differences were based on P < 0.05.

## Additional Information

**How to cite this article**: Park, J. H. *et al*. Variation in structure of proteins by adjusting reactive oxygen and nitrogen species generated from dielectric barrier discharge jet. *Sci. Rep.* **6**, 35883; doi: 10.1038/srep35883 (2016).

## Supplementary Material

Supplementary Information

## Figures and Tables

**Table 1:** Possible reactions and rate constants for the Argon-oxygen plasma.

Reactions	Rate Constant (cm^3^/s)	Ref.
e + O_2_ → 2O + e (1)	4.2 × 10^−9 ^e^−5.6/Te^	29,30
O + O + M → O_2_ + M (2)	5.2 × 10^−35^e^900/T^	29,30
e + O_2_ → O^−^ + O (3)	8.8 × 10^−11^ e^−4.4/Te^	29,30
e + O_2_ → O_2_^+^ + 2e (4)	9.0 × 10^−10^ T_e_ e^−12.6/Te^	29,30
e + O^−^ → O + 2e (5)	2.0 × 10^−7^ e^−5.5/Te^	29,30
O^−^ + O_2_^+^ → O + O_2_ (6)	2.0 × 10^−7^ (300/T)^0.5^	29,30
O^−^ + O → O_2_ + e (7)	2.0 × 10^−10^	29,30
O + O_2_ + M → O_3_ + M (8)	1.9 × 10^−35^ e^1057/T^	30,31
O_3_ + M → O + O_2_ + M (9)	7.3 × 10^−10^ e^−11400/T^	30,31
