# Supplementary material for: Variation in structure of proteins by adjusting reactive oxygen and nitrogen species generated from dielectric barrier discharge jet
Source: Sci Rep. 2016 Oct 25;6:35883. doi: 10.1038/srep35883 (PMC5078802; doi:10.1038/srep35883)
Supplement: Supplementary Information [file srep35883-s1.pdf]

Supporting information

Variation in structure of proteins by adjusting reactive oxygen and nitrogen species generated from dielectric barrier discharge jet

Ji Hoon Park[1#], Minsup Kim[2#], Masaharu Shiratani[3], Art. E. Cho[2]*, Eun Ha Choi[1]*, Pankaj Attri[1,3]*[#]

[1]Plasma Bioscience Research Center/Department of Electrical and Biological Physics, Kwangwoon University, Seoul 01897, Korea.
[2]Department of Bioinformatics, Korea University, Sejong 02841, Korea.
[3]Graduate School of Information Science and Electrical Engineering, Kyushu University, Fukuoka, Japan.

Figure Captions

Figure S1: OES spectra of (a) Ar ; (b) Ar-O_2 (0.4% O_2) and (c) Ar-N_2 (0.4% N_2).

Figure S2: Change in (a) Temperature and (b) pH after DBD jet treatment for 4 min using feeding gases such as pure Ar, Ar-O_2 and Ar-N_2 at different ratios.

Figure S3: Fluorescence spectra of (a) Hb and (b) Mb at different time interval treatment

Figure S4: Fluorescence spectra of Hb treatment at different time interval and in different Ar-O2 ratios (a) 0.2% O_2; (b) 0.3% O_2 and (c) 0.4% O_2

Figure S5: Fluorescence spectra of Hb treatment at different time interval and in different Ar-N2 ratios (a) 0.2% N_2; (b) 0.3% N_2 and (c) 0.4% N_2

Figure S6: Fluorescence spectra of Mb treatment at different time interval and in different Ar-O2 ratios (a) 0.2% O_2; (b) 0.3% O_2 and (c) 0.4% O_2

Figure S7: Fluorescence spectra of Mb treatment at different time interval and in different Ar-N2 ratios (a) 0.2% N_2; (b) 0.3% N_2 and (c) 0.4% N_2

Figure S8: FTIR spectra for 0.4% O_2 and 0.4% N_2 admixture to Ar plasma for different time interval (a) Hb at 1650 cm^{-1}; (b) Hb at 1630 cm^{-1}; (c) Mb at 1650 $cm^{-1}$and (c) Mb at 1630 cm^{-1}

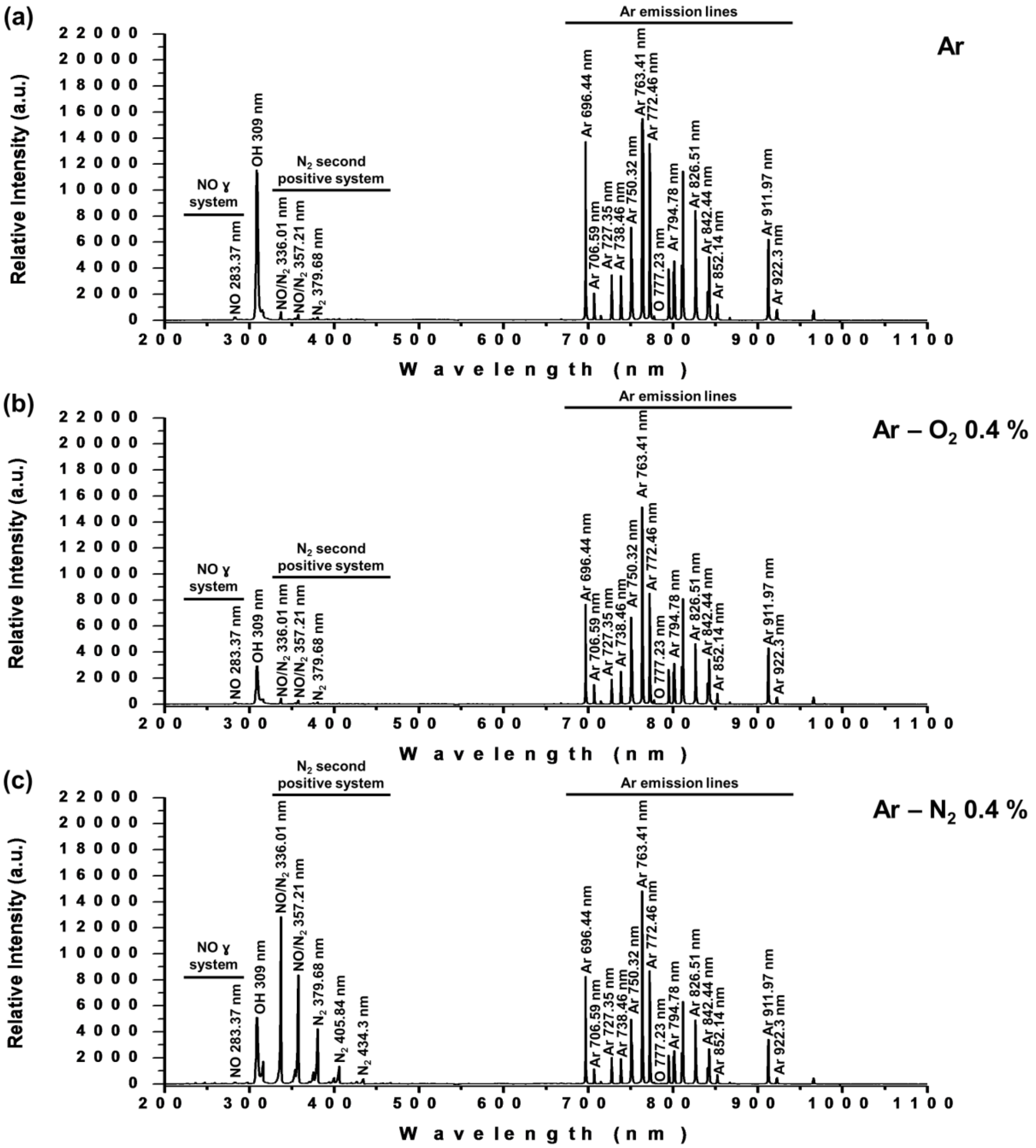
(a)
Ar
Ar emission lines
NO γ system
N$_2$ second positive system
NO 283.37 nm
OH 309 nm
NO/N$_2$ 336.01 nm
NO/N$_2$ 357.21 nm
N$_2$ 379.68 nm
Ar 696.44 nm
Ar 706.59 nm
Ar 727.35 nm
Ar 738.46 nm
Ar 750.32 nm
Ar 763.41 nm
Ar 772.46 nm
O 777.23 nm
Ar 794.78 nm
Ar 826.51 nm
Ar 842.44 nm
Ar 852.14 nm
Ar 911.97 nm
Ar 922.3 nm
Relative Intensity (a.u.)
Wavelength (nm)
(b)
Ar – O$_2$ 0.4 %
(c)
Ar – N$_2$ 0.4 %
N$_2$ 405.84 nm
N$_2$ 434.3 nm

Figure S1

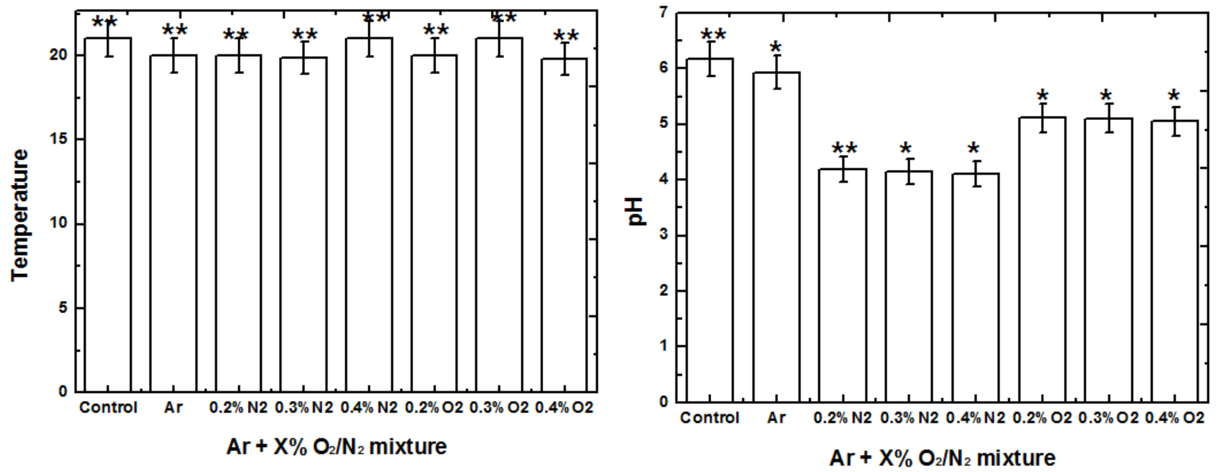

Figure S2

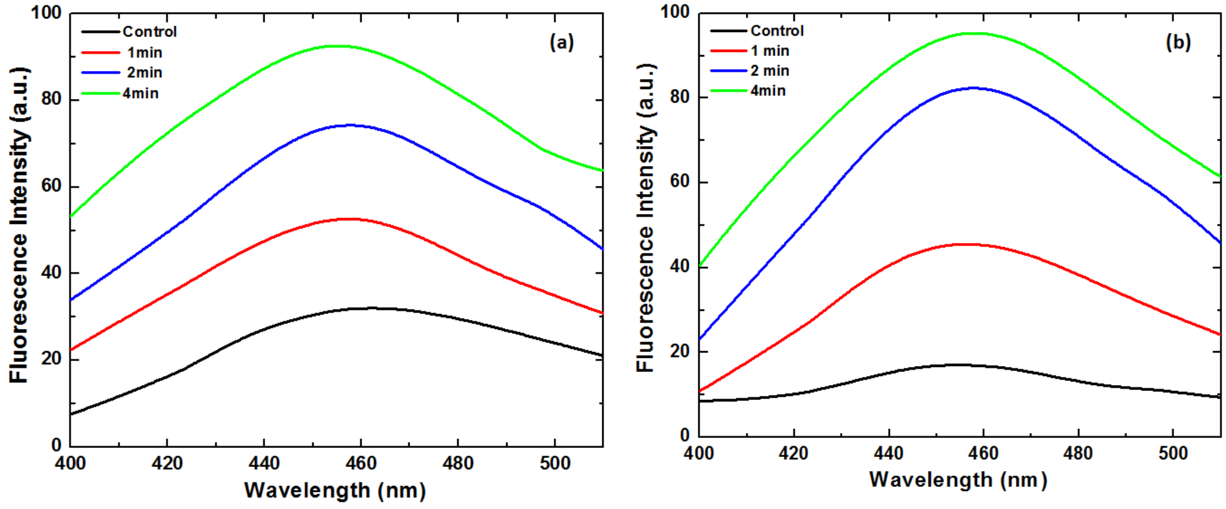

Figure S3

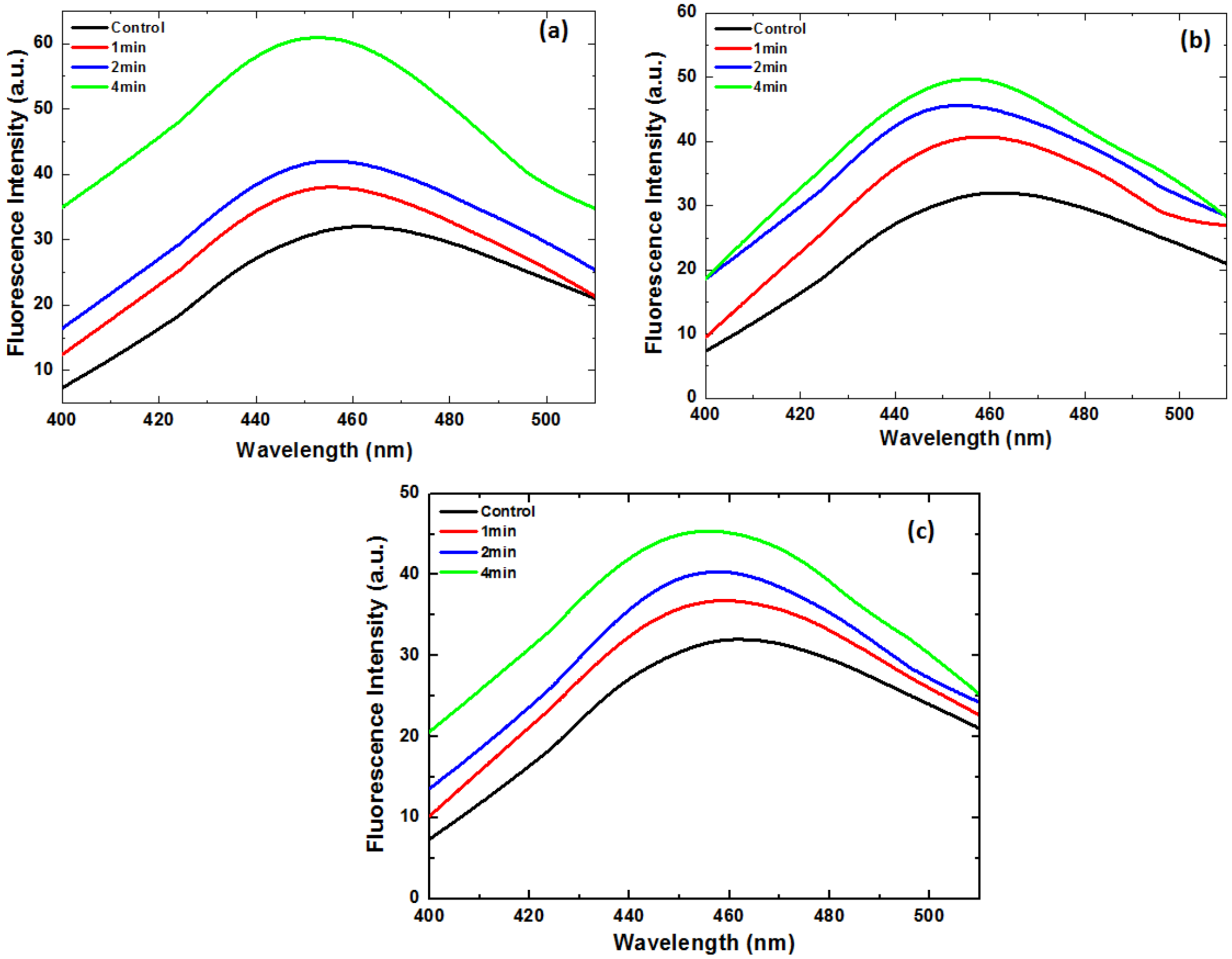
(a)
(b)
(c)
Control
1min
2min
4min
Fluorescence Intensity (a.u.)
Wavelength (nm)
400
420
440
460
480
500
0
10
20
30
40
50
60

Figure S4

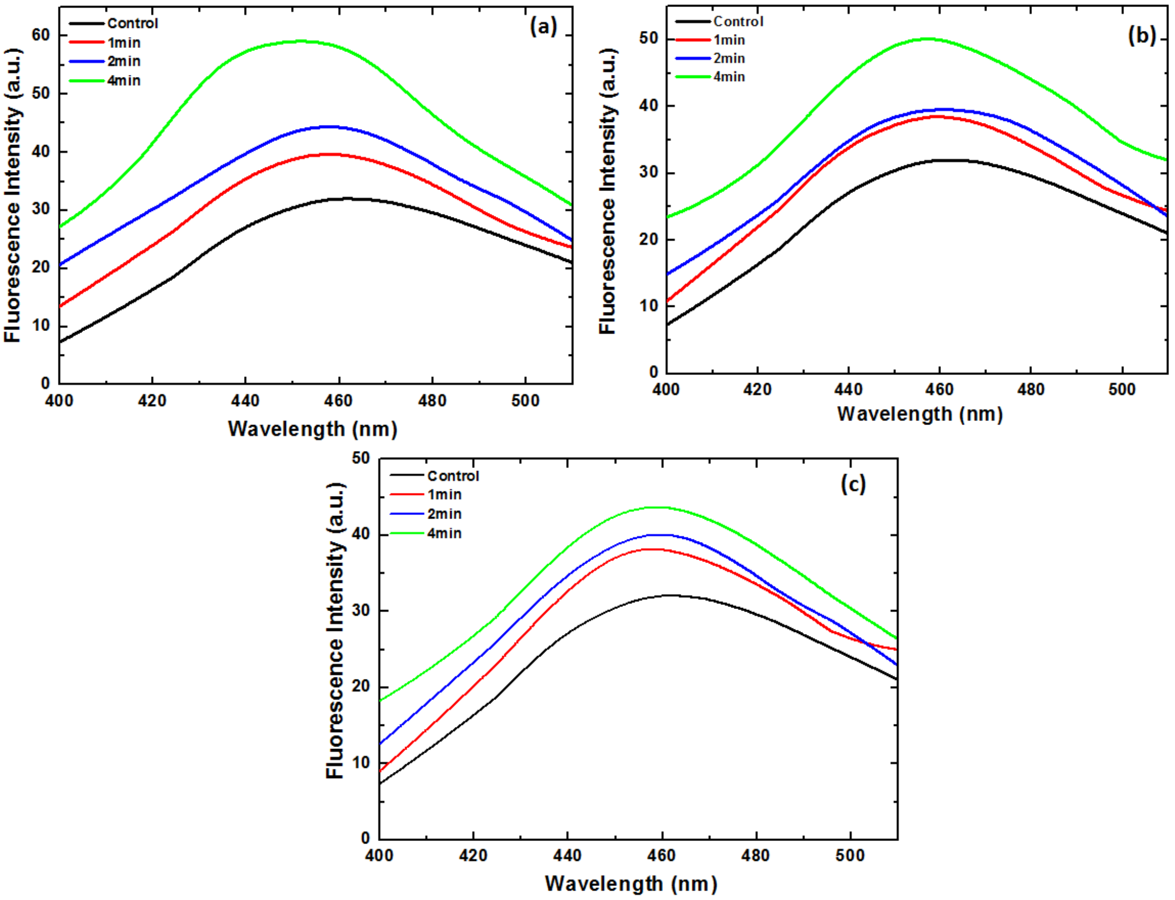
Fluorescence Intensity (a.u.)
Wavelength (nm)
Control
1min
2min
4min
(a)
(b)
(c)

Figure S5

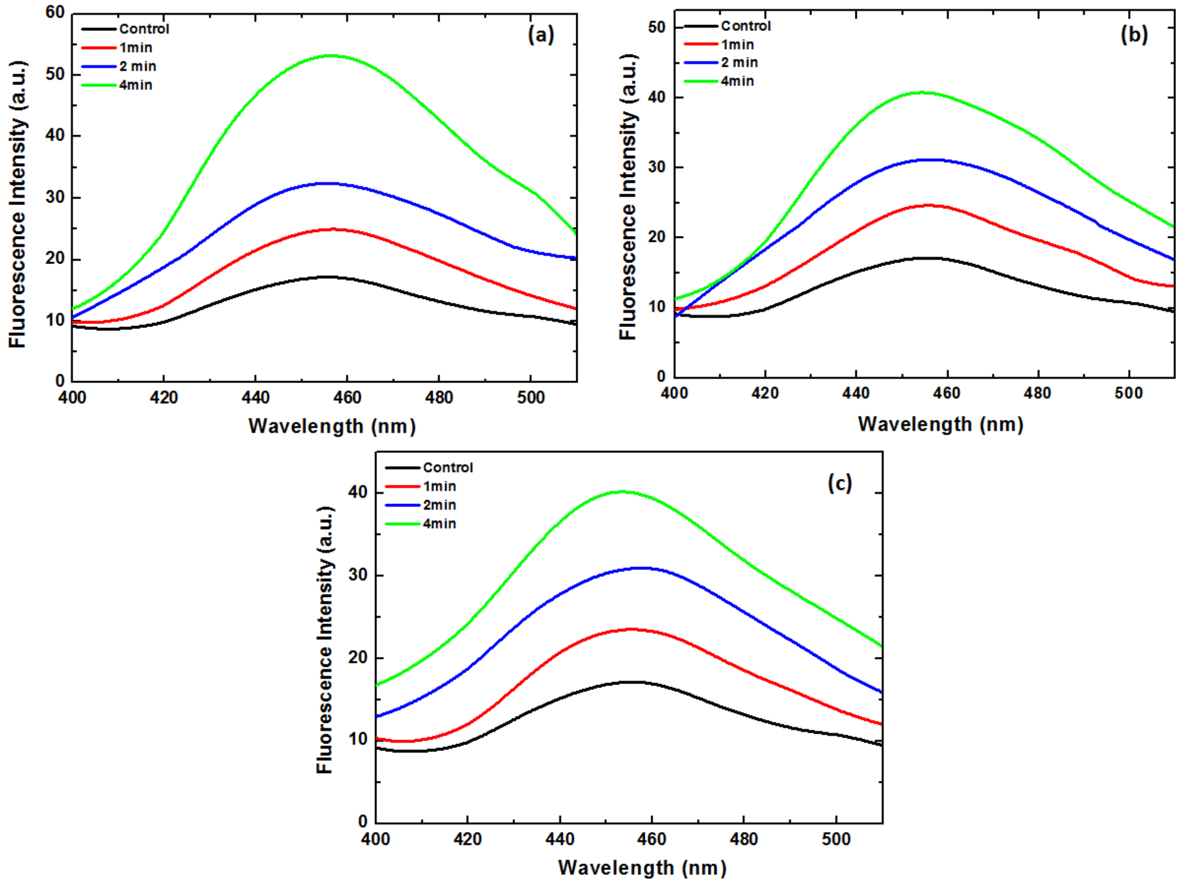
(a)
Control
1min
2 min
4min
Fluorescence Intensity (a.u.)
Wavelength (nm)
0
10
20
30
40
50
60
400
420
440
460
480
500
(b)
Control
1min
2 min
4min
Fluorescence Intensity (a.u.)
Wavelength (nm)
0
10
20
30
40
50
400
420
440
460
480
500
(c)
Control
1min
2min
4min
Fluorescence Intensity (a.u.)
Wavelength (nm)
0
10
20
30
40
400
420
440
460
480
500

Figure S6

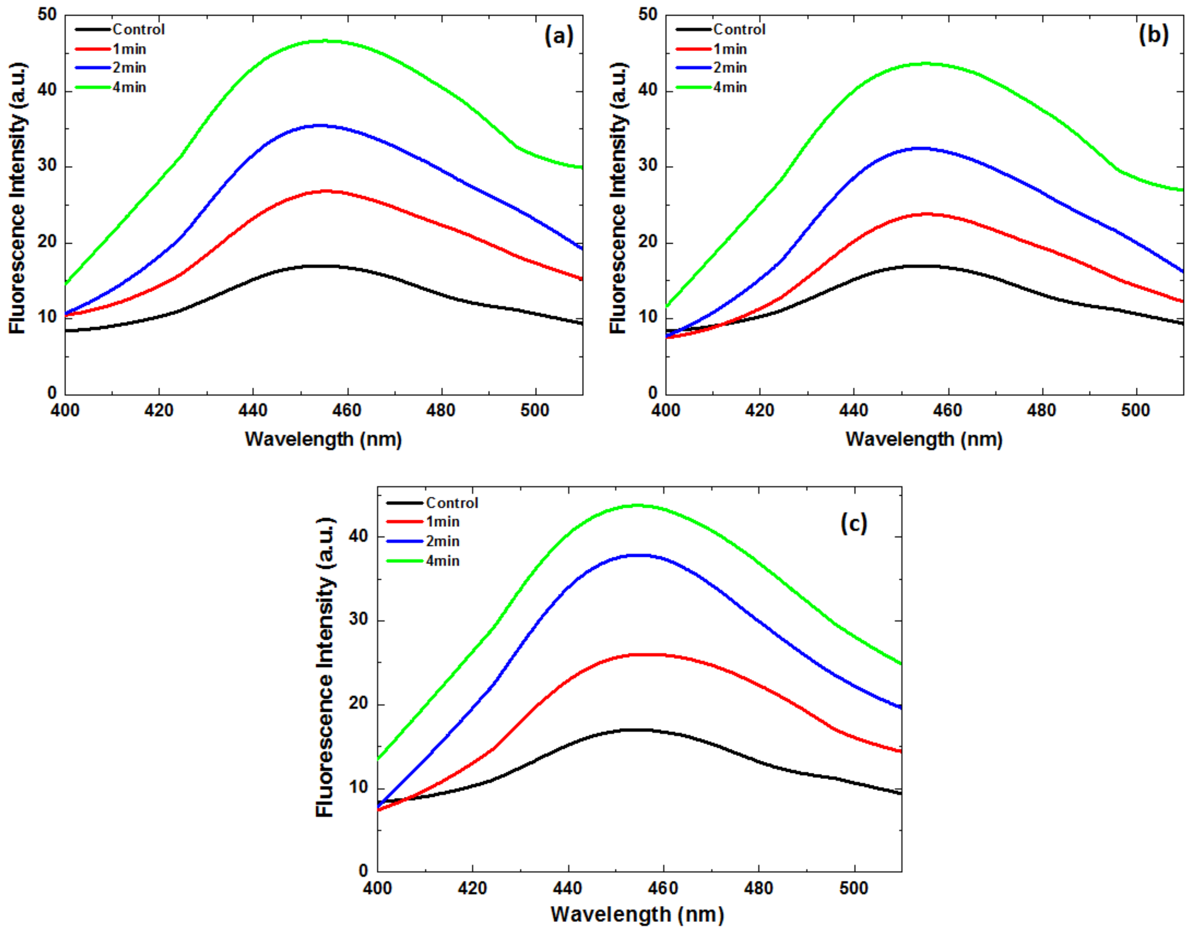

(a)
(b)
(c)
Control
1min
2min
4min
Fluorescence Intensity (a.u.)
Wavelength (nm)
0
10
20
30
40
50
400
420
440
460
480
500

Figure S7

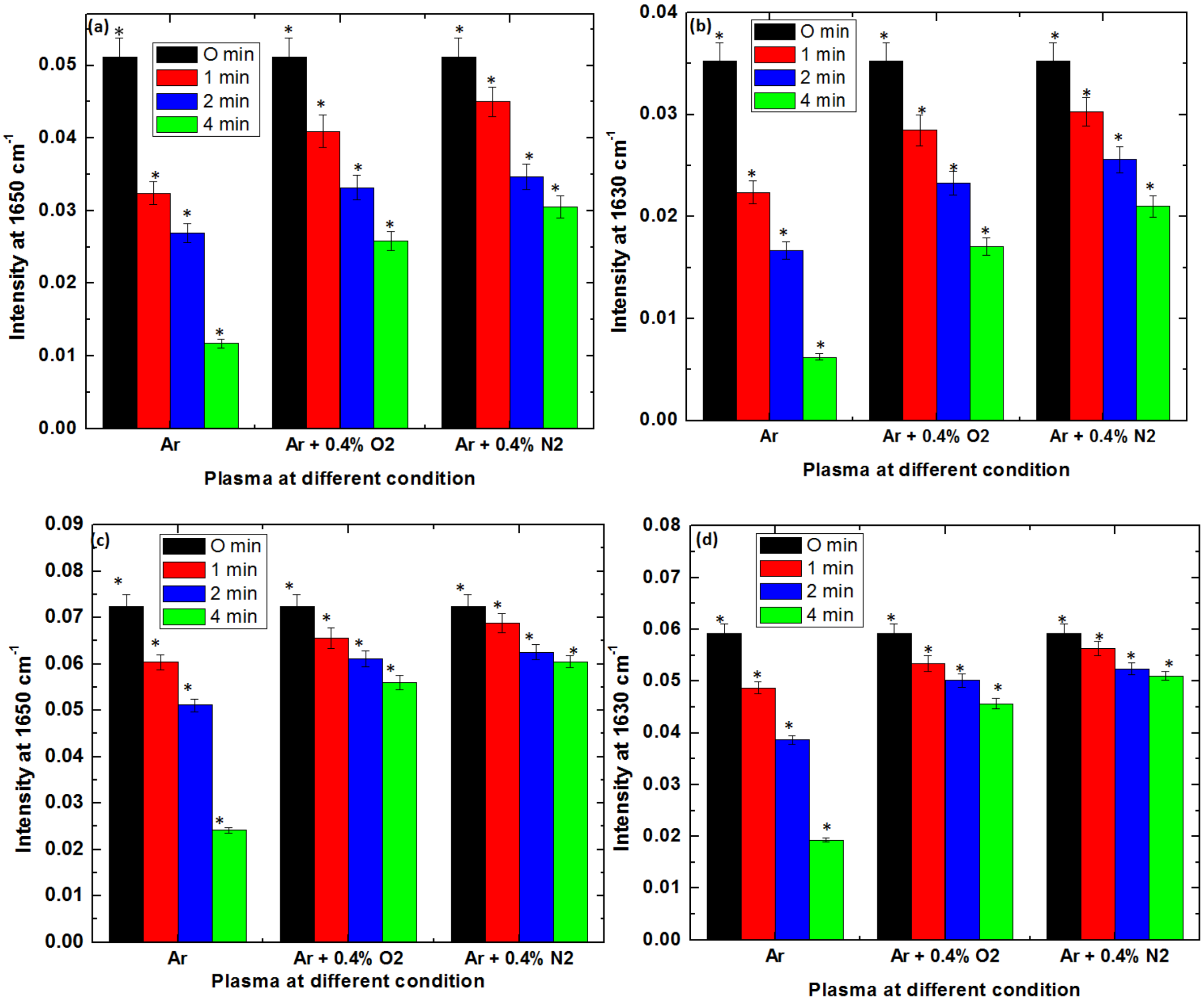
(a)
O min
1 min
2 min
4 min
Intensity at 1650 cm-1
Ar
Ar + 0.4% O2
Ar + 0.4% N2
Plasma at different condition
(b)
Intensity at 1630 cm-1
(c)
(d)

Figure S8
